# From neurons to novelty: Circuit mechanisms shaping courtship evolution

**DOI:** 10.1016/j.conb.2025.103137

**Published:** 2025-11-15

**Authors:** Philipp Brand, Rory T. Coleman

**Affiliations:** 1 Evolutionary Neuroscience and Behavior Group, Max Planck Institute for Brain Research, Frankfurt am Main, Germany; 2 Department of Biology, Center for Neural Science, New York University, New York, NY, USA

## Abstract

The vast diversity of animal behaviors has long inspired ethologists and neuroscientists, but circuit mechanisms driving this variation remain elusive. Recent advances in genetic tools and comparative approaches have enabled unprecedented insights into how neural circuits evolve to produce behavioral novelty. Here we focus on the discoveries emerging from the study of courtship behaviors, which are particularly well poised to capitalize on these advances. Comparative studies of sensory and motor circuits have begun to demonstrate that evolution can act through diverse mechanisms. The modular organization of courtship-controlling circuits emerges as a key feature facilitating rapid evolutionary innovation while maintaining essential functions. Changes in the neuronal composition of circuits, by both cellular and subcellular mechanisms, represent common mechanisms. Organisms may even carry vestigial circuits with the latent potential to be repurposed for new behavioral paradigms. We highlight how understanding the ‘extended nervous system’ of a species has begun to provide these critical insights into courtship evolution and offers fertile ground for future discoveries. As comparative approaches expand beyond model organisms, evolutionary neuroscience is on the cusp of revealing the principles governing behavioral diversity in nature.

Among the most striking characteristics of metazoans are the extraordinary diversity and complexity of their behavioral feats. Yet remarkably little is known about how this behavioral diversity arises from evolutionary adaptations within the nervous system. While studies on conventional model organisms have revealed fundamental aspects of neurobiology and circuit function, the evolutionary distance separating worms, flies, rodents, fish, and apes makes it difficult to identify where and how evolution acts on circuits to shape behavior. Similarly, the work of ethologists and evolutionary biologists has catalogued remarkable patterns of behavioral evolution, but proximate mechanisms remain elusive due to a lack of incisive genetic tools that would allow access to the underlying circuitry. The genome engineering revolution now presents an unprecedented opportunity to synthesize these approaches and glean new insights into the governing principles that shape how neural circuits and the behaviors they organize diverge. The ability to begin to compare the neuroanatomy and function of homologous circuits in phylogenetically close but behaviorally divergent species offers the potential to begin to link proximate and ultimate mechanisms, a goal Tinbergen laid out as essential to a holistic understanding of the biology of animal behavior [1].

Of the rich repertoire of animal behaviors, those surrounding mating offer a powerful system in which to approach this problem. In particular, courtship rituals are extraordinarily diverse across species, while at the same time highly stereotyped within species, an excellent prerequisite to begin to dissect neural changes causing behavioral variation. Further, the sexual dimorphism inherent to courtship, and its underlying genetic basis, has proven an invaluable means to identify the underlying circuits, providing easy access to neural substrates and simplified comparisons between male and female brains [2,3]. Naturalistic courtship is often reliably ported into laboratory conditions, allowing for reductionist experiments to probe component behaviors and their underlying circuitry that are nonetheless interpretable in the context of ethology [4,5]. These features have facilitated combining cross-species comparisons of homologous neural circuits with the ethology of courtship behaviors, allowing for the identification of neural underpinnings of variation in courtship strategies and thus accommodating the fundamental goals of neuroscience and evolutionary biology alike. While neuroscience is focused on the function of the nervous system, evolutionary biology tasks itself with identifying the causes and consequences of variation, but both are essential to an integrated understanding of how behaviors diverge. A mechanistic comparative approach offers an opportunity for a union within the realm of evolutionary neuroscience.

Here we offer an introduction to courtship as a model behavioral paradigm for evolutionary neuroscience, review recent advances in our understanding of the evolution of courtship behaviors, and discuss the principles underlying behavioral diversification and nervous system function that have begun to emerge (see Refs. [6,7] for reviews on broader evolutionary circuit principles). We argue that the concept of an ‘extended nervous system’ provides a powerful framework to illuminate the evolution of behavior through the lens of sexual dimorphism in courtship. Finally, we point out intriguing avenues for future advancement.

## Courtship evolution: from multimodal sensory integration to complex motor output

**Figure 1:**
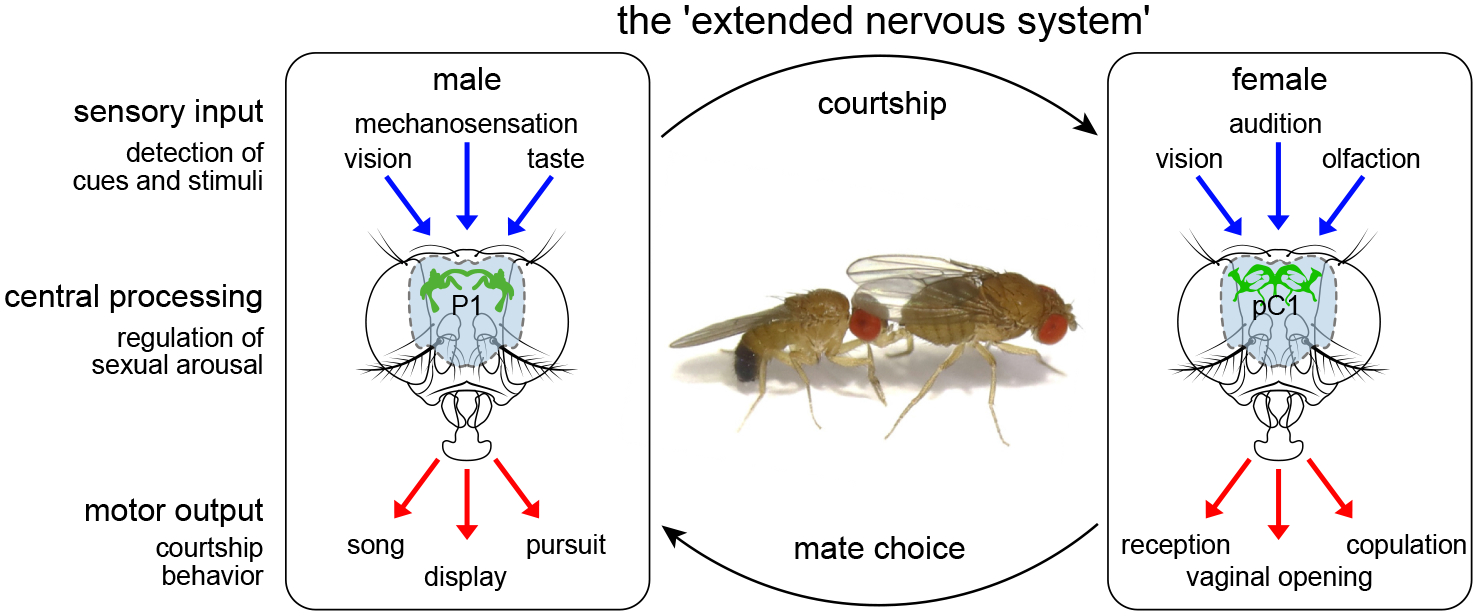
The extended nervous system. To understand the evolution of courtship behaviors, it is not sufficient to focus on the nervous system of the courting individual, often the male, but to consider the ‘extended nervous system’ of both parts of the courting pair. Courtship behaviors use and act themselves as signals aimed to match the sensory preferences of the opposite sex. In return, the courted individual responds, requiring coordination between courter and target highlighted using *Drosophila* as example. Without knowledge of the ‘extended nervous system’ eco-evolutionary trajectories of courtship behavior cannot be fully derived.

In animals, courtship comprises the set of behaviors one individual displays to attract a conspecific mating partner of the opposite sex for reproduction. These range from simple behaviors, including the release of an attractive long-range sex pheromone in moths, to elaborate courtship routines such as those exhibited by bowerbirds, where males build complex structures from gathered materials and perform impressive visual and acoustic displays to attract females. A major outstanding question is how and what parts of the nervous system facilitate the evolution of such staggering diversity in courtship rituals. While courtship behaviors are typically elaborated in only one sex, often males, they act as signals aimed at matching the sensory preferences of the opposite sex. Indeed, courtship is often composed of successive, interdependent interactions between the courter and the target meaning that some degree of coordination is required for successful copulation [8–10]. Accordingly, it is not sufficient to focus on the nervous system of the courting individual, but to consider the **‘extended nervous system’** (Figure 1), which we define as constituting the neural circuits of both parts of the courting pair. When viewing courtship evolution from this integrated framework, two major aspects of the extended nervous system must be considered: (1) the evolution of courtship signals and their perception and (2) innovations in motor programs.

## Signals and signal perception

Courtship signaling has been a classic focus of ethology and evolutionary biology, identifying how signaling traits such as coloration and pheromone chemistry evolve to maximize attraction to the opposite sex. Examination of the coevolutionary relationship between display trait diversification and signal preference has delivered compelling insights into the underlying drivers of behavioral innovation. Signal and receiver components are expected to co-evolve to ensure successful matching between conspecific males and females (reviewed in Ref. [11]). Distinct evolutionary mechanisms have been identified, classified by the directionality of adaptation and the environmental context during trait and preference diversification. Sensory exploitation, where a courtship trait evolves to match a pre-existing sensory preference in the opposite sex is one such example. In the frog *Physalaemus pustulosus*, for instance, males evolved lower frequency ‘chuck’ courtship calls to match the conserved auditory profile of females of their species, thereby ‘exploiting’ their predisposed sensory preference [12]. Environmental conditions can also play an important role in shaping courtship display evolution. Male coloration in *Pundamilia* cichlids has evolved towards the red-shifted light spectrum visible in the deeper turbid waters of Lake Victoria, following the change in opsin tuning driven by the environmental conditions, a mode of signaling evolution called sensory drive [13,14]. In addition to these examples where adaptation is well explained by distinct mechanisms, it is likely that courtship evolution is driven by a combination of sexual and non-sexual selective pressures. In Hawaiian cricket populations for example, a novel courtship ‘humming’ has been discovered to offer a fitness advantage, because it acoustically stimulates conspecific females while going undetected by parasitoid flies unlike the ancestral courtship song [15]. These studies deliver compelling neurophysiological explanations for functional mechanisms driving courtship trait evolution by examining the tuning of sensory organs involved in the receiving sex. Indeed, most examples of neural evolution underlying courtship behaviors have been linked to variation in the sensory periphery [16]. While these findings exemplify one of the likely many mechanisms of trait reception evolution, they often are not sufficient to fully explain preference evolution. Further, the weight of evidence pointing to peripheral mechanisms likely reflects an availability bias resulting from the significant challenges associated with studying neural variation in higher order brain centers.

Technical advances in connectomics, single cell transcriptomics, volumetric imaging, and genome engineering promise to soon level this bias, as central circuits become increasingly accessible to cross-species approaches (see Future directions). As a result, new mechanistic insights into the neural variation underlying courtship evolution beyond the sensory periphery have begun to emerge. For the time being, these studies are primarily based on cross-species comparisons centered around model organisms such as *Drosophila melanogaster* [17], due to the wealth of neurogenetic tools available that can now be translated to closely related species.

In Drosophilids, as in other insects [18], pheromone cues play a primary role in directing courtship toward appropriate targets [19,20], whereas visual information serves as a non-discriminatory courtship promoting cue, as evinced by the sufficiency of fly-sized dots or beads to promote courtship [21–23]. Consistent with this, pheromonal signaling has diverged rapidly across the genus, with multiple instances of independent convergence on the same chemical pheromones [24–27]. Such natural evolutionary experiments offer unique opportunities for comparison across repeated evolutionary trajectories — when female courtship signals converge, do male courtship circuits co-evolve along predictable paths or in idiosyncratic ways instead?

**Figure 2:**
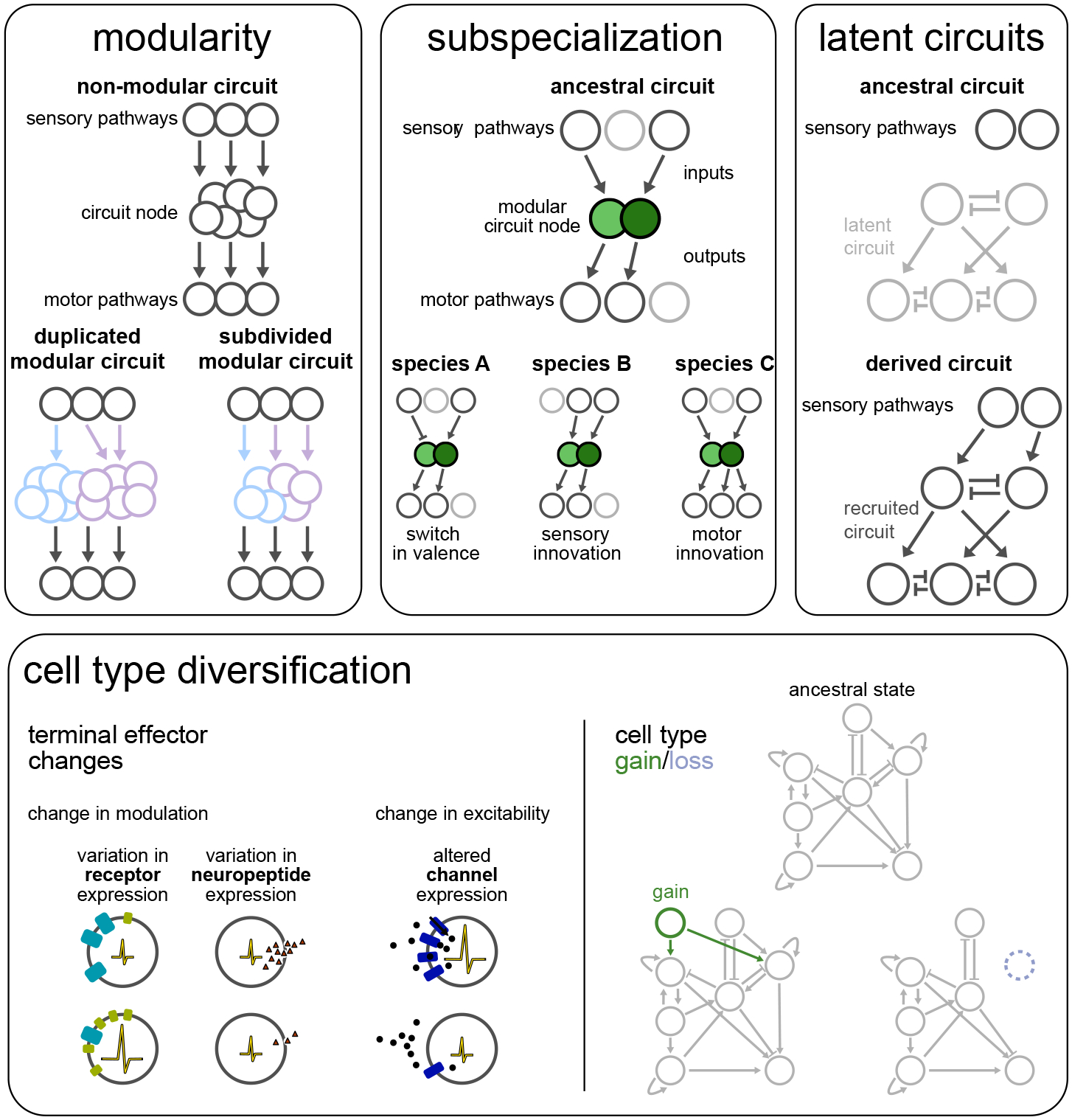
Emerging principles of courtship circuit evolution. Cross-species comparative analyses have revealed several neural principles underlying the evolution of courtship behaviors. Duplication of neuronal populations or molecular subdivision of preexisting neuronal population through altered developmental patterning mechanisms leads to modular circuit architectures. Modular circuit architecture facilitates flexible behavioral diversification via variation in sensory input and possibly motor output. Such organization also allows the engagement of latent circuits. A variety of cellular and subcellular mechanisms contribute to courtship diversification. See main text for details and references.

Surprisingly, investigation into how male pheromone perception co-evolved with changes in female pheromone production between the sister species *D. melanogaster* and *D. simulans* revealed conservation in pheromone processing at the level of sensory detection. Instead, differences in pheromone perception results from circuit adaptations at the level of a conserved population of P1 neurons in the central brain that regulate a male’s sexual arousal state [28]. Another recent study again found changes in processing at the level of the central P1 neurons to be a key step in the diversification of male pheromone preference in the species *D. yakuba* [29]. In *D. yakuba*, however, central changes were coordinated alongside additional diversification in the tuning of sensory neurons. Given the rich examples of peripheral diversification underlying behavioral evolution, and the recent discovery of central circuit diversification as a key factor in the evolution of pheromone processing, linked changes in both peripheral and central circuits can perhaps be expected to be what is most typical. If such disseminated circuit modifications are the norm, however, it raises an important question: how can multi-level changes be accommodated in a way that maintains coherent circuit function? Early indications suggest that the **modular organization of circuits** within the brain is a key feature of rapidly evolving male courtship circuits (Figure 2).

Strong evidence for the key role of modular circuit architectures in courtship evolution comes from the dissection of the neurons that constitute the P1 node in *Drosophila*. P1 neurons are organized into several morphologically and genetically distinct subtypes [2,30,31], which receive input from separate sensory pathways. The studies of *D. simulans* and *D. yakuba* demonstrate that the sensory signals converging at P1 are variable across species [28,29]. This variation in patterns of integration appears to be driven by different P1 subtypes, which act as flexible receivers of sensory inputs [29]. Thus, the central P1 courtship node represents a flexible evolutionary switchboard where single elements (i.e. subtypes) are free to diversify while others retain ancestral function, similar to what has been proposed for regions of the vertebrate brain that display comparable properties [32,33]. Thus, the redundancy inherent to such a modular system presumably relieves constraints and allows evolution to tinker with sensory inputs, an idea that has been put forward as a central aspect of degeneracy in the nervous system [34,35]. Accordingly, redundant courtship control nodes, like the subtypes that constitute P1, are unconstrained and free to **subspecialize**, resulting in changes in who, when, or where an animal engages in courtship (Figure 2). In addition to sensory input, such modular organization may also pattern variation in motor output downstream. This would, for example, allow existing behavioral motifs to be recruited into the new context of courtship or alter redundant motifs.

One proposed process through which modular circuit architectures might arise is via duplication of cell types, neural populations, or even entire circuits [36–38] (Figure 2). While modularity might be an emergent property in certain contexts [39], duplication events offer a plausible mechanism for rapidly relieving the pleiotropic constraints pronounced in central circuit architectures, allowing evolution to tinker with multiple duplicated units. Indeed, circuits across the animal tree of life are thought to be highly degenerate [40,41], suggesting that multiplication of cells and circuits is a common feature of brain evolution. This mechanism resembles the process of gene evolution by duplication and subsequent sub- or neofunctionalization [42–44]. While at first approximation, neural expansions may seem evolutionarily more complicated compared to the singular molecular event that produces duplicated genes, duplication of lineage-related neurons may result from similarly discrete mutations [45]. Such a mechanism was proposed as the origin of the vocal learning pathway of songbirds [46]. Alternatively, modularity could emerge through changes in developmental specification, leading a previously homogeneous neural population to be subdivided into heterogeneous molecular identities due to changes in patterning cues such as morphogens (Figure 2). In all likelihood, both duplication and subdivision events have been sources of neural modularity across circuits and the tree of life. However, the extent to which neural duplication is a source of modularity in mating circuits remains an important hypothesis to be explored.

## Motor output

While the fundamental role of sensory systems in controlling the timing and context of behaviors bears enormous evolutionary potential for the diversification of courtship, sensory processing alone is insufficient to explain the extent of variation observed in nature. Divergence in courtship song, dance and other forms of display — prominent parts of mating rituals in many insects, birds, and mammals — must require changes in downstream motor organizing pathways. Understanding the neural underpinnings creating behavioral variation therefore requires not only the examination of circuits controlling the recruitment but also the execution of motor programs. These circuits are usually highly complex. Bird song alone requires the coordination of many, individually innervated muscles, which does not include those that control dance or plumage display, which often co-occur simultaneously during courtship. Beginning to dissect such complicated motor organizing systems remains a persistent challenge, but systems-level analysis in genetically tractable species offers a promising inroad.

Courtship songs have proven a useful model to study the evolution of motor output and the associated effects on reproduction (see Ding & Lillvis, this issue). Collectively, such studies have pointed to neural **cell type diversification**—through changes in the cell type makeup of circuits or in terminal effectors of neuronal identity [47] (Figure 2). In *Drosophila*, male flies create songs by vibrating their wings when courting, a sound that is highly arousing to conspecific females. The resulting sexual selection on these courtship songs has led them to rapidly diversify to guide mate choice. Importantly, the motor organizing circuit that coordinates this song has recently been delineated from the *D. melanogaster* connectome [48], providing a roadmap for identifying sites of diversification. Comparative analyses of *D. melanogaster* and *D. yakuba* found that an anatomically and physiologically conserved population of song command neurons, pIP10, produces highly distinctive songs in the two species [49], indicating the recruitment of divergent downstream motor organizers. Indeed, loss of a specific song type in *D. yakuba*, known as sine song, is associated with changes in the innervation of the wing neuropil located downstream of pIP10. Single-cell sequencing together with anatomical dissection revealed that TN1A neurons, necessary for organizing sine song in *D. melanogaster*, have been significantly reduced or lost in *D. yakuba* largely through programmed apoptosis [50]. This is a remarkable example of adaptive cell type loss in the evolution of a species-specific courtship behavior and is consistent with other cross-species examinations that suggest that species-specific differences in patterns of apoptosis are likely wide-spread and potential contributors to behavioral variation [51,52]. A similar phenomenon by a distinct mechanism has been observed in the *Xenopus* clade, where activation of motor organizers in *ex vivo* preparations produces distinct male advertisement calls in *X. laevis* compared to *X. petersii*. The species specificity in *Xenopus*, however, appears to be a consequence of intrinsic membrane properties, possibly due to changes in ion channel expression, rather than a structural change in the circuit [53,54] (Figure 2). Cell-type-diversification driven changes in courtship strategy are not restricted to motor circuits. In *Heliconius* butterflies, *H. cydno* males prefer to court females according to wing pigmentation patterns. This color preference appears to be at least partially attributable to variation in the strength of inter-photoreceptor inhibitory connections [55]. More thorough investigation of pathway diversification, aided by the rise of connectomics, will offer the opportunity to identify the full range and prevalence of mechanisms that evolution utilizes when innovating on both sensory and motor programs.

While comparative investigation of courtship circuits in males has become increasingly tractable, variation in females has been largely neglected. Nonetheless initial insights suggest similar mechanisms may be involved in the evolution of female reproductive behaviors. Wing spreading is a unique behavior that *D. santomea* females elicit in response to male courtship and is required for mate acceptance and copulation. Changes in conserved sexually dimorphic descending neurons controlling the adaptive wing movement have been implicated as the likely circuit mechanism [9]. Activation of homologous groups of descending neurons in *D. santomea* and *D. melanogaster,* which does not naturally exhibit wing-spreading, resulted in spread wings in both species. These findings suggest that an ancestral circuit has the **latent potential** to recruit variable downstream motor organizers to control diverse courtship behaviors across species (Figure 2).

Interestingly, the activation of sexually dimorphic neurons expressing the sex determination gene *doublesex* in *D. melanogaster* females leads to the expression of male courtship behaviors, indicating that females retain a latent circuit for organizing male courtship motor programs despite never recruiting them in nature [56] (Figure 2). These observations suggest that latent circuits might represent a potent substrate for evolution to shape behaviors. Their detection, however, can be exceedingly difficult and requires further investigation. That the few places where such latent circuits have been identified were in the context of cross-sex and cross-species comparative approaches highlights the necessity of studying the extended nervous system of an organism across meaningful axes of variation.

## Future directions

Recent advances in transgenics have significantly accelerated the study of nervous system function and anatomy across closely related species. While principles are beginning to emerge across lineages, these have largely been based on model organism-centered studies. It will be necessary to branch out into diverse groups throughout Metazoa to test the validity of these emergent evolutionary patterns. Circuit-level studies of courtship evolution across species remain especially biased, because most of our knowledge is derived from work centered around *D. melanogaster*. However, the recent surge in comparative neuroscience has allowed scientists to investigate the neural underpinnings of species differences in a diverse set of behaviors including feeding in nematodes [57,58], parental care [59] and threat avoidance in deer mice [60], swimming in nudibranchs [61], and sexually dimorphic behaviors across flies [62], indicating that the necessary techniques are increasingly available to study behavioral evolution — including courtship — across a wide range of clades. A growing number of non-model organisms are becoming increasingly genetically tractable, allowing for the development of neurogenetic toolkits in previously inaccessible species such as the translucent fish *Danionella translucida* [63] or mosquitoes [64,65], which is a prerequisite for future cross-species examination. Similarly, electrophysiological methods have been adapted to record neural activity in diverse vertebrate (e.g. tufted titmouse: [66]; Egyptian fruit bat: [67]; chain catshark: [68]) and invertebrate species (paper wasp: [69]; octopus: [70]), together indicating that neuroscience is at the brink of an era that will enable a growing number of studies comparing functional properties of brain areas, circuits, and neurons across species, further integrating ethology, neuroscience, and evolutionary biology.

So far the circuit-level investigation of courtship evolution has mainly focused on neural function and high-level anatomy. To further advance our understanding, it will be necessary to study behavioral variation from additional perspectives. A major outstanding question is how neural variation underlying behavioral differences is encoded genetically, an important question especially when studying instinctive behaviors such as courtship that do not require learning to execute in most animals.

The rapid development of single-cell sequencing approaches, which has accelerated the identification of cell type evolution and its role in behavioral diversification [37,59,71], holds promise for insight into how cell types evolve, either through population level gains or losses, or more subtle changes in the molecular identities of neurons (Figure 2). Connectomics, a rapidly growing field in brain anatomy that aims at describing the connections between all neurons of a nervous system at synaptic resolution, will help link how genetic shifts in cellular identity relate to fine-scale changes in circuit structure and function. Several whole-brain connectomes of distantly related animals have been made accessible to the scientific community with increasing pace (worm: [72], fly [73,74]: reviewed in Ref. [75]). Recently, the first detailed comparative-connectomics studies in nematodes, flies, and mammals have been reported [76–78], providing an exciting glimpse into what will likely become a key approach in the future investigation of behavioral evolution. It is only a matter of time before connectomes combined with molecular profiling by single-cell sequencing or MERFISH [79] between closely related species will offer broad support for the identification and further interrogation of homologous courtship circuits and their evolution across species. Early work using single-cell RNA-sequencing approaches has begun to identify genetic fingerprints of cell types involved in courtship across species, suggesting that this is indeed a promising future direction [50,80]. Finally, current rapid technical and methodological advances in functional imaging and AI promise to further accelerate the study of behavioral diversification, suggesting a wide range of tools that will be available for future investigations of courtship evolution.

## Conclusion

A circuit-level understanding of behavioral evolution is only beginning to emerge, aided by advances in genetic techniques that allow for cross-species comparison of homologous neural circuits. The resulting budding field of evolutionary neuroscience has revealed new insights into the divergence of neural circuits that underlies the evolution of courtship behaviors. While currently biased towards *Drosophila*, accessibility of circuits in various animal groups is increasing, promising exciting developments in the future. Technological advancements in multiple areas of neuroscience should facilitate studies of behavioral evolution into many other branches of the animal tree of life, opening previously inaccessible clades with amazing behavioral diversity for deep interrogation. We think there is no better time than now to partake in the rapidly growing field of evolutionary neuroscience.

## Data Availability

No data was used for the research described in the article.
